# Antineoplastic activity of a nutrient mixture in Y-79 malignant retinoblastoma cells

**DOI:** 10.3892/or.2012.2110

**Published:** 2012-10-29

**Authors:** M. WAHEED ROOMI, NUSRATH ROOMI, BILWA BHANAP, ALEKSANDRA NIEDZWIECKI, MATTHIAS RATH

**Affiliations:** Dr Rath Research Institute, Cancer Division, Santa Clara, CA 95050, USA

**Keywords:** Y-79 cells, retinoblastoma, matrix metalloproteinase, apoptosis, nutrient mixture, invasion

## Abstract

Retinoblastoma is one of the most common ocular malignancies in children under the age of six. Occasionally, retinoblastoma metastasizes to extraocular organs including the bone, lung and brain. Left untreated, retinoblastoma is fatal. At present, there is no effective treatment for metastatic retinoblastoma. We investigated the antineoplastic activity of a nutrient mixture (NM) (lysine, proline, ascorbic acid and green tea extract) at concentrations of 10, 50, 100, 500 and 1,000 μg/ml in triplicate at each dose in the human malignant retinoblastoma Y-79 cell line. The parameters used were cell proliferation, expression of matrix metalloproteinases (MMPs), invasion through Matrigel, morphology and apoptosis. Cell viability was assessed by trypan blue dye exclusion test. Invasion was evaluated through Matrigel and MMP activity by gelatinase zymography. H&E staining for morphological cell alterations and apoptotic studies using the Live Green Poly Caspase Detection kit were also conducted. The nutrient mixture at 10–100 μg/ml demonstrated approximately 25% toxicity towards Y-79 retinoblastoma cells and significant toxicity at 500 and 1,000 μg/ml. The Y-79 cells secreted only MMP-2 as demonstrated by zymography; the nutrient mixture had no effect on MMP-2 expression up to 100 μg/ml, but completely blocked it at 500 μg/ml. Importantly, Y-79 retinoblastoma cells were not invasive through Matrigel. H&E staining showed cell morphological changes related to apoptosis, which was confirmed using the Live Green Poly Caspase Detection kit. Our results suggest that this nutrient mixture, which inhibited cell proliferation, expression of MMP-2 and induced apoptosis, may be a candidate for further exploration for its therapeutic potential in metastatic retinoblastoma.

## Introduction

Retinoblastoma is a malignant tumor of the retina of the eye and generally affects children under the age of six years. Retinoblastoma is rare and it affects approximately 1 in 15,000 live births. Worldwide, approximately 5,000 new cases occur per year, while in the US that incidence is 300 cases/year (1). It is the most common eye cancer in children and is caused by mutation on chromosome 13, called the RB1 gene. The defective RB1 gene can be inherited from either of the parents in some children; however, the mutation occurs in the early stages of fetal development. Characterized by the typical cat’s eye or the white pupil reflex (leukocoria) noted by parents, approximately 63% of all retinoblastomas arise in the first two years of life. In some cases, retinoblastoma metastasizes to extraocular organs including bone, lung and brain. Although non-metastatic tumors can be treated by enucleation (removal of the eye), currently, there is no treatment for metastatic retinoblastoma (1). Only 10% of cases tend to have a family history. Retinoblastoma gene has been identified as an abnormality on chromosome 13. Parents with a familial bilateral retinoblastoma have 50% chance of passing it on to their children. In addition, sporadic mutation in the gene can still be passed on to the next generation even though the parent did not inherit the gene or suffer any cancer because of it. Ninety percent of the children who develop retinoblastomas are the first ones in their families to have it. The survival rate drops with each decade of life for patients with a genomic mutation (2,3).

Cancer cells from tumors spread by degrading the extracellular matrix (ECM) with the use of a group of endopeptidase enzymes, the matrix metalloproteinases (MMPs). Activity of these enzymes correlates with the aggressiveness of tumor growth and metastasis. In 1992, Rath and Pauling (4) postulated that nutrients such as lysine and ascorbic acid act as natural inhibitors of ECM proteolysis and as such have the potential to modulate tumor growth and metastasis. These nutrients can exert their antitumor effect both through inhibition of MMPs and strengthening the connective tissue surrounding cancer cells (tumor encapsulating effect). In previous *in vitro* and *in vivo* studies, we demonstrated the antitumor potential of a nutrient mixture (NM) in a number of cancer cell lines (5–7).

Considering the efficacy of NM on other cancer cell lines, we investigated the effects of NM on the Y-79 retinoblastoma cell line regarding cell proliferation, modulation of MMP expression, cell invasive potential by Matrigel invasion and apoptosis and cell morphological changes using the Live Green Poly Caspase Detection kit and H&E staining, respectively.

## Materials and methods

### Composition of the nutrient mixture (NM)

Stock solution of the NM prepared for testing was composed of the following: vitamin C (as ascorbic acid and as magnesium, calcium and palmitate ascorbate) 700 mg; L-lysine 1,000 mg; L-proline 750 mg; L-arginine 500 mg; N-acetylcysteine 200 mg; standardized green tea extract 1,000 mg (green tea extract was derived from green tea leaves obtained from US Pharma Lab). The certificate of analysis indicates the following characteristics: total polyphenol 80%, catechins 60%, epigallocatechin gallate (EGCG) 35% and caffeine 1.0%; selenium 30 μg; copper 2 mg; manganese 1 mg.

### Cell culture

The retinoblastoma Y-79 cell line was obtained from the American Type Culture Collection (ATCC, Manassas, VA). The cells were cultured on Roswell Park Memorial Institute (RPMI)-1640 medium containing 20% fetal bovine serum and antibiotics. The cells were grown in a humidified 5% CO_2_ atmosphere at 37°C and later treated with the NM at 0, 10, 50, 100, 500 and 1,000 μg/ml in triplicate at each dose. Cells were also treated with Phorbol 12-myristate 13-acetate (PMA) to induce MMP secretion. The plates were then returned to the incubator.

### Cell proliferation study

Cell proliferation was assessed by trypan blue dye exclusion test after 24 h, as previously described (8). Viable cell count was expressed as a function of the control.

### Gelatinase zymography

MMP secretion in conditioned media was determined by gelatinase zymography as previously described (9). In brief, gelatinase zymography was performed in 10% polyacrylamide Novex^®^ precast gel, sodium dodecyl sulphate (SDS) (Invitrogen Corp.), in the presence of 0.1% gelatin under non-reducing conditions. Culture medium (20 μl) was loaded and SDS-polyacrylamide gel electrophoresis (SDS-PAGE) was performed with Tris-glycerine SDS buffer as described by the manufacturer (Novex). Samples were not boiled before electrophoresis. After electrophoresis, the gels were washed with 5% Triton X-100 for 30 min at room temperature to remove SDS. The gels were then incubated at 37°C overnight in the presence of 50 mM Tris-HCl, 5 mM CaCl_2_, 5 μM ZnCl_2_ at pH 7.5, stained with Coomassie Blue R 0.5% for 30 min and destained. Protein standards were run concurrently, and approximate molecular weights were determined by plotting the relative mobilities of known proteins.

### Matrigel invasion studies

Invasion studies were conducted using Matrigel (Becton-Dickinson) inserts in 24-well plates (9). In brief, the malignant retinoblastoma Y-79 cells suspended in medium were supplemented with nutrient, as specified in the design of the experiment and seeded on the insert in the well. Thus, both the medium on the insert and in the well contained the same supplements. The plates with the inserts were then incubated in a culture incubator equilibrated with 95% air and 5% CO_2_ for 24 h. After incubation, the media from the wells were withdrawn. The outer surface of the insert was washed gently and the media and washing were collected in the well. The media were spun, and the cells were counted.

### Assessment of cell morphology

Cell morphology of the cells cultured for 24 h in the test concentrations of NM was evaluated by H&E staining and observed for apoptotic changes and images were captured.

### Analysis of apoptosis

Apoptosis was determined by the method described in the Live Green Poly Caspase Detection kit at different doses of NM. Cells were challenged with NM at concentrations of 0, 50, 100, 250, 500 and 1,000 μg/ml and incubated for 24 h. The culture was washed with PBS and treated with caspase reagent as specified in the manufacturer’s protocol (Molecular Probes Image-IT™ Live Green Poly Caspase Detection kit 135104; Invitrogen Corp.). Cells were photographed under a fluorescence microscope and counted. Green-colored cells represented viable cells, while yellow-orange-colored cells represented cells undergoing early apoptosis and red-colored cells represented those undergoing late apoptosis.

### Statistical analysis

The results are expressed as means ± standard deviation (SD) for the groups. Data was analyzed by the independent sample t-test.

## Results

### MTT study

**Figure 1:**
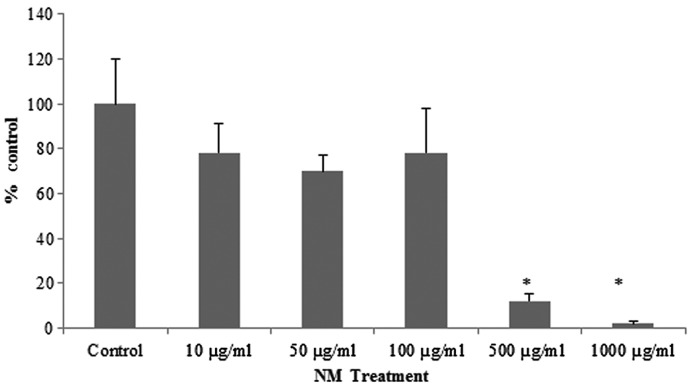
Retinoblastoma Y-79 cells exhibit gradually increasing toxicity after treatment with NM. ^*^Significant at p=0.002.

NM treatment of the retinoblastoma Y-79 cells resulted in 25% toxicity at NM doses of 10–100 μg/ml. However, significant toxicity was observed in the cells exposed to concentrations of 500 and 1,000 μg/ml NM (Fig. 1).

### Gelatinase zymography study

**Figure 2:**
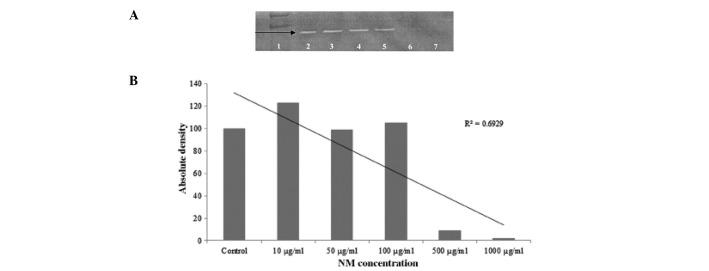
MMP-2 expression and analysis. (A) Gelatinase zymography revealed expression of MMP-2 in the retinoblastoma Y-79 cells. The expression was completely blocked following exposure at a concentration of 500 μg/ml NM. 1, marker; 2, control; 3–7, 10–1,000 μg/ml NM). PMA treatment did not induce MMP-9 expression. (B) Densitometric analysis demonstrated relative activity of MMP-2 after treatment with NM at doses of 10–1,000 μg/ml.

Gelatinase zymography study revealed only one band corresponding to MMP-2. PMA treatment did not induce MMP-9 expression. The expression of MMP-2 was not affected by NM up to 100 μg/ml. However, it was significantly inhibited at an NM concentration of 500 μg/ml with virtually total inhibition at 1000 μg/ml (Fig. 2A). This was further confirmed by densitometric analysis as shown in Fig. 2B, with the R^2^ value being 0.6929.

### Cell invasion studies

The malignant retinoblastoma Y-79 cells did not exhibit invasiveness through Matrigel (data not shown).

### Analysis of cell morphology (H&E staining)

**Figure 3:**
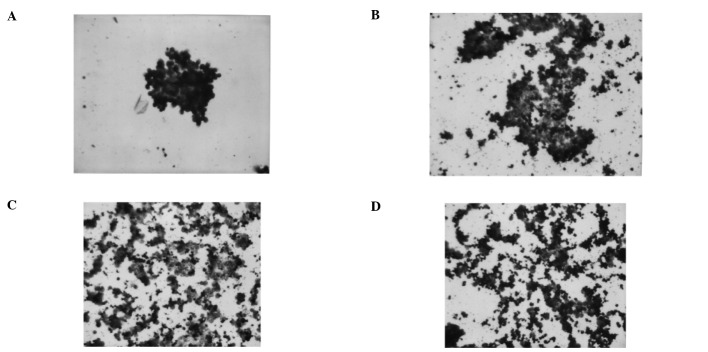
NM treatment results in gradually increasing toxicity to Y-79 cells as noted by cell morphology. (A) Control, (B) 100 μg/ml NM, (C) 500 μg/ml NM; (D) 1,000 μg/ml NM.

H&E staining demonstrated obvious cell apoptosis. Apoptotic cells showed shrinkage with deeply stained and condensed nuclei and strongly acidophilic cytoplasm (Fig. 3A-D).

### Detection of apoptosis

**Figure 4:**
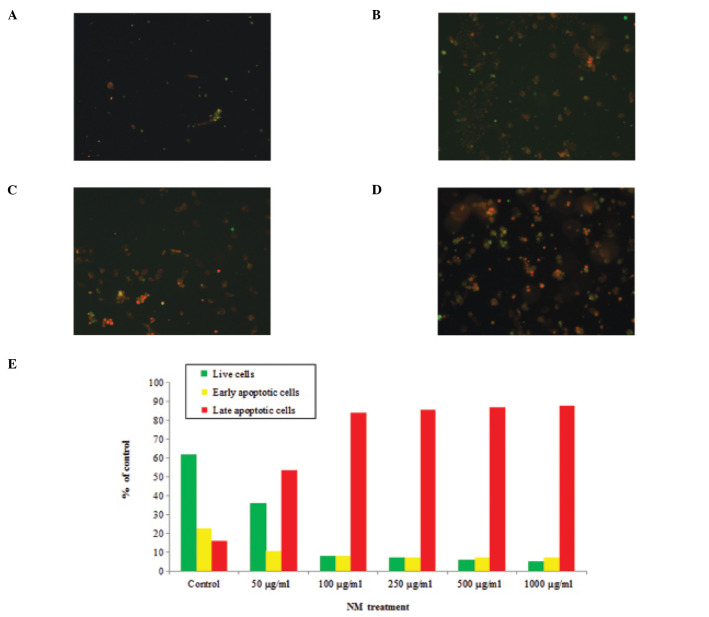
Effect of NM on the apoptosis of Y-79 cells using the Live Green Poly Caspase kit. NM induced apoptosis of retinoblastoma cells in a dose-dependent manner. (A) Control, (B) 50 μg/ml NM, (C) 250 μg/ml NM, (D) 1,000 μg/ml NM. (E) Quantitative analysis of living, early and late apoptotic Y-79 cells revealed a dose-dependent effect with increasing NM doses. Apoptosis was evident with 61% living cells and 16% late apoptotic cells noted following exposure of 50 μg/ml NM. Following treatment with increasing concentrations of NM, the percentage of late apoptotic cells gradually increased up to 88%, while the percentage of living cells decreased accordingly to 5% at 1,000 μg/ml.

Apoptosis was assessed using the Live Green Poly Caspase Detection kit. Dose-dependent apoptosis of Y-79 cells was evident following NM challenge (Fig. 4A–D). A moderate amount of cell apoptosis was observed in cells exposed to 50 μg/ml NM. However, the extent of apoptosis increased significantly with increasing doses of NM up to 1,000 μg/ml. Quantitative analysis of living, early and late apoptotic cells is shown (Fig. 4E). Following exposure to 50 μg/ml NM, the percentage of viable Y-79 cells was observed to be 36% and that of late apoptotic cells was 53%. With increasing concentrations of NM, the percentage of late apoptotic cells gradually increased up to 88%, the percentage of early apoptotic cells was 7%, while the percentage of living cells decreased to 5% following treatment with 1,000 μg/ml of NM.

## Discussion

In the present study, we investigated the effects of NM on malignant retinoblastoma cell line Y-79. The results indicated that NM had a profound inhibitory effect on the proliferation of these cells and inhibition of MMP secretion. NM also induced apoptosis. These are the most important steps in cancer metastasis.

Free radical injury plays a key role in cancer initiation and progression. During the multistep process, the degradation of ECM by MMPs is a critical step in tumor growth, invasion and metastasis. It is important to restrict this step to halt tumor progression. Ascorbic acid, a potent antioxidant, used alone has been shown to have a cytotoxic effect on Y-79 cells (10,11).

Aggressiveness of retinoblastoma is highly correlated with the expression of MMPs, which by degrading surrounding ECM contributes to the invasiveness of cancer. Although the relationship of MMP enzymes to cancer progression and metastasis has been studied in many types of cancer, their importance in retinoblastoma has not been established until recently. Researchers have recently demonstrated that MMP-2 activity is directly involved in the differentiation of retinoblastoma cells. They concluded that, ‘therapeutics targeting to MMP-2 may prove useful for reducing malignancy through the differentiation of retinoblastoma cells’ (12). In our study, we demonstrated that the expression of MMP-2 enzymes can be completely blocked by NM at a concentration of 500 μg/ml. Although in our study the Y-79 cells did not secrete MMP-9, the expression of this enzyme has been considered to directly contribute to the cellular proliferative process in retinoblastoma. Based on the recent understanding of the importance of MMP enzymes in retinoblastoma it has also been suggested that differential expression of MMP-9 and MMP-2 could be a significant pathologic factor reflecting the biology of retinoblastoma and may also be used as a monitoring test.

The results of our MTT assay and apoptosis studies demonstrated that NM has profoundly toxic effects on Y-79 cells. Many of our previous studies with various cancer cell lines have shown the anticancer effects of NM to be mediated through ECM stability. This study indicates that the NM effectiveness in Y-79 cells was through a pro-apoptotic effect. This effect appears to be cancer-specific since our previous studies demonstrated no NM toxicity to a variety of normal cells, such as fibroblasts, smooth muscle cells and endothelial cells (13,14).

In the attempt to understand the etiology of retinoblastoma, researchers have explored factors such as parental age, occupation, exposure to toxins and maternal nutrition. The etiology of sporadic retinoblastoma linked to the maternal diet and nutrition has been suggested (15). Deficiencies in nutrients in the first year of life also appear to contribute to the genetic mutation in retinoblastoma. Based on our current results we believe that the dietary supplementation of NM should be explored further both in preventive and therapeutic aspects of retinoblastoma and its metastasis.
